# Do HBsAg subdeterminants matter for vaccination against hepatitis B?

**DOI:** 10.1007/s11262-024-02061-y

**Published:** 2024-02-14

**Authors:** Wolfram H. Gerlich

**Affiliations:** Institute of Medical Virology, Justus Liebig University Giessen, National Reference Center for Hepatitis B and D, Schubert Str. 81, Giessen, D35392 Germany

In the current issue of *Virus Genes*, a case of chronic hepatitis B infection (CHB) is described in a young woman who was vaccinated in early childhood against hepatitis B (HB) during the vaccination campaign which was started in Egypt during the 1990s [1].

## The case

Although being just a case report, this failure of protection by the vaccination is noteworthy because the infecting hepatitis B virus (HBV) strain had two unexpected substitutions in the so-called “a” determinant in comparison to the HBV subgenotype D1 which is predominant in Egypt and in the entire Middle East. The vaccine was most likely the widely used “recombinant” HB vaccine ENGERIX-B which consists of the small hepatitis B surface antigen (HBsAg) expressed in genetically modified yeast. According to the vaccination program in Egypt, the infants were given three doses at 2, 4, and 6 months of age. A birth dose was not given routinely, but a screening of pregnant women for HBsAg was usually done. The high efficacy of this vaccination program has been proven in Egypt as only 14 (0.39%) of 3600 vaccinated children aged 0.8 to 16 years showed signs of an HBV infection, and only 4 (0.11%) of these had a CHB with persistent HBsAg [2]. The 20-year-old patient described in this manuscript had no protective level of anti-HBs, was HBsAg positive, HBV DNA moderately high positive with 1500 IU/ml, anti-HBc positive, IgM anti-HBc negative, and had no signs of liver disease. This pattern suggested a CHB with low activity. CHB develops usually after being infected perinatally or in early childhood and rarely later in life if no immune deficiency exists.

## The vaccine escape strain

The two identified amino acid (AA) substitutions T125M and P127T were within the HBsAg AA sequence 124–147 forming the determinant “a” [3]. The authors concluded that the HB vaccination had probably induced an anti-HBs response, and this may have caused a selection of the mutant or the mutations within the infecting HBV subgenotype D1. This conclusion is compatible with the fact that the patient did not have anti-HBs antibodies anymore 20 years after vaccination. The evaluation of the HB vaccination campaign in Egypt [2] showed that only 30.5% of the > 15-year-old adolescents were still anti-HBs positive while vaccinated infants at the age of 1 year were 93% positive. However, there is no data suggesting that the protection against infection HBV subgenotype D1 should have initially failed and that the anti-HBs response should have caused selection of an HBV strain with these two major mutations.

## HBsAg determinants and HBV genotypes

The two AA substitutions T125M and P127T hinted to the alternative possibility that the patient was infected early in life by an unusual HBV subgenotype D9 first described in India [4] which is characterized by these two AA substitutions; this is in contrast to most other subgenotypes of genotype D. If this was the case, no mutations at all would have been selected in the HBsAg gene. However, subgenotype D9 infections had not yet been identified in Egypt. The source of the HBV infection of the patient is unknown but Salama and coworkers [2] mention numerous medical interventions in the HB vaccinated children they studied. How could the HB vaccination possibly have favored infection with this rare HBV subgenotype? Here, the antigen determinants and subdeterminants of HBsAg come into play [5, 6]. AA position 127 in the HBsAg sequence is not only part of the determinant “a” but it furthermore defines the HBsAg subdeterminants w_1_-w_4_. Determinant “a” was defined 51 years ago as the antigenic site common to all HBsAg samples. Virtually all HBsAg samples showed two additional pairs of mutually exclusive antigenic sites called “d” or “y” and “w” or “r”. Thus, an HBsAg sample could have the subtype designation adw, ayw, adr or ayr. The determinants “d/y” and “w/r” could easily be separated from determinant “a”, because “d/y” were encoded by the polymorphisms at AA122 (K for “d” and R for “y”) and AA160 (K for “w” and R for “r”). Soon after, subdeterminants of w, w_1_-w_4_ were discovered but it took a further 19 years to identify the AAs defining these subdeterminants. AA P127 forms w_1_ and w_2_, AA T127 forms w_3_ and AAs I/L127 form w_4_ [5]. With today’s knowledge, it would be more adequate to designate these subdeterminants a_1 − 4_ instead of w_1 − 4_ [6].

## HBsAg subdeterminants and HB vaccines

Present day HB vaccines are still based on the cloning, sequencing and expression of the gene encoding the HBsAg in the 1970s. Not surprisingly, one of the first two cloned HBs genes had the subtype formula adw_2_ (or a_2_d) associated with the HBV subgenotype A2 predominant in USA; this gene is still used for production of the most widely used HB vaccine, e.g., ENGERIX-B. It is plausible to assume that the rare HBV subgenotype D9 with the HBsAg subtype formula a_3_y (or ayw_3_) could more easily evade the probably existing early anti-HBs response of the patient than HBV subgenotype D1 which shares the subdeterminant a_2_ (or w_2_) with the HB vaccine. A study in U.S. blood donors [7] showed that recently acquired HBV infections in vaccinated donors were caused significantly more often by non-A2 subgenotypes with various (sub)determinants (C2, adr; F1, a_4_d; B2, a_2_d?; D, ay; D, ay) than in non-vaccinated donors which were all infected with A2 (a_2_d). Four vaccinated subjects were infected by HBV strains with HBsAg (sub)determinants different from the HB vaccine, suggesting that the (sub)determinants may be important for immune protection.

## “a”-subdeterminants and natural immune selection

The subdeterminants a_1 − 4_ are defined by AA127. Evidence is accumulating that AA127 and the neighboring AAs have a key role in anti-HBs immune protection against HBV, induced either by vaccination or natural immune responses. The P127T substitution causes a switch from a_2_ to a_3_ and has been mentioned in a review on HB vaccine escape [3]. A particularly drastic example of immune selection by naturally induced anti-HBs leading to change or complete loss of the subdeterminant a_3_ was recently described [8]. A 53 old CHB patient with low HBV DNA and coincident HBsAg and anti-HBs had been infected by HBV subgenotype D3 with the wildtype (sub)determinant a_3_y (T127). During 20 years of CHB, four different mutated strains were selected with a_4_y (T127L), a_2_y (T127P), and two unspecified a_x,y_ subdeterminants with the AA substitutions T127R and T127V. Fourteen further AA substitutions were described between AA125-134 in these four mutants, eight of them generating new N-glycosylation sites; this underlines the strong selective immune pressure of anti-HBs antibodies on this part of the “a” determinant including the a_2 − 4_ subdeterminants.

## Conclusion

The HBV HBsAg subdeterminants a_1 − 4_ have largely been neglected up to now, but they deserve more attention for the diagnosis of HBV infections and for the development of protective HB vaccines.
